# ADHD, Lead, and PCBs: Eubig et al. Respond

**DOI:** 10.1289/ehp.1103513R

**Published:** 2011-07-01

**Authors:** Paul A. Eubig, Andréa Aguiar, Susan L. Schantz

**Affiliations:** Department of Comparative Biosciences, College of Veterinary Medicine, University of Illinois at Urbana-Champaign, Urbana, Illinois, E-mail: eubig@illinois.edu

In response to Brondum’s comments we would like to reiterate that the main purpose of our review (Eubig et al. 2010) was to examine the parallels between cognitive domains affected in children with attention deficit/hyperactivity disorder (ADHD) and domains shown to be affected in human and animal studies of developmental exposure to lead and polychlorinated biphenyls (PCBs), two environmental contaminants for which a relatively large body of literature exists. In doing so we hoped to explore the possible role of exposure to environmental contaminants in the variable phenotypic expression of ADHD, and to stimulate interest in further research in this area.

In our review, we did not seek to identify individual behavioral tests or functional domains that could serve as surrogates for ADHD diagnosis. Nor did we make the case that developmental lead and PCB exposure are responsible for the rise in ADHD diagnoses in recent years. To the contrary, in our section on other environmental contaminants we specifically highlighted the fact that PCB and lead exposures are declining, whereas exposures to other chemicals—including brominated flame retardants, bisphenol A, phthalates, polyfluoroalkylated compounds and certain pesticides—are increasing. We note that studies of the potential role of these emerging contaminants in the etiology of ADHD are equally, if not more, important than further studies of lead and PCBs. In addition, there is a clear difference between exploring contaminants as potential contributors to ADHD risk as opposed to causing ADHD. Examining our Table 6 (Eubig et al. 2010), which showed a comparison of the strength of the evidence for cognitive domains affected in ADHD with domains affected in developmental lead and PCB exposure, should convince the reader that these three conditions are similar but not the same. Brondum seems to miss this point in implying that our review is without value because the studies that evaluated the association between lead and a diagnosis of ADHD, which comprise a relatively small part of our review, are flawed in his opinion.

No one is debating whether parental psychopathology should be considered as a possible confounding factor in studies that examine the association of contaminant exposure with specific neurobehavioral diagnoses, including ADHD. Braun et al. (2007) acquiesced to that point in a reply to the first of several letters to the editor by Brondum (2007) on the same topic. However, Braun et al. (2007) noted that including such information is not always possible when, for example, the use of National Health and Nutrition Examination Survey (NHANES) data or other constraints on study design do not allow it. Although we agree that failure to control for parental psychopathology is a weakness in many of the published studies reporting an association between childhood lead exposure and a diagnosis of ADHD, we believe that the consistency of the association across several published studies using different study designs adds to the weight of evidence that this is a real association and not a spurious effect due to uncontrolled confounding.

We hope that this reply clarifies the goals of our review for those faced with the challenge of assessing the neurobehavioral effects of emerging contaminants and their possible contribution to the phenotypic expression of ADHD or other neurodevelopmental disorders.

## References

[r1] Braun JM, Lanphear BP, Kahn RS, Froehlich T, Auinger P (2007). ADHD: Braun et al. Respond [Letter]. Environ Health Perspect.

[r2] Brondum J. (2007). Environmental Exposures and ADHD [Letter]. Environ Health Perspect.

[r3] Eubig PA, Aguiar A, Schantz SL (2010). Lead and PCBs as risk factors for attention deficit/hyperactivity disorder.. Environ Health Perspect.

